# Humidity- and Temperature-Tunable Metal–Hydrogel–Metal Reflective Filters

**DOI:** 10.1021/acsami.1c15616

**Published:** 2021-10-13

**Authors:** Semyon Chervinskii, Ibrahim Issah, Markus Lahikainen, Alireza R. Rashed, Kim Kuntze, Arri Priimagi, Humeyra Caglayan

**Affiliations:** Faculty of Engineering and Natural Sciences, Tampere University, 33720 Tampere, Finland

**Keywords:** metal−insulator−metal, hydrogel, tunable color filter, stimuli-responsive material, active plasmonics, PNIPAm

## Abstract

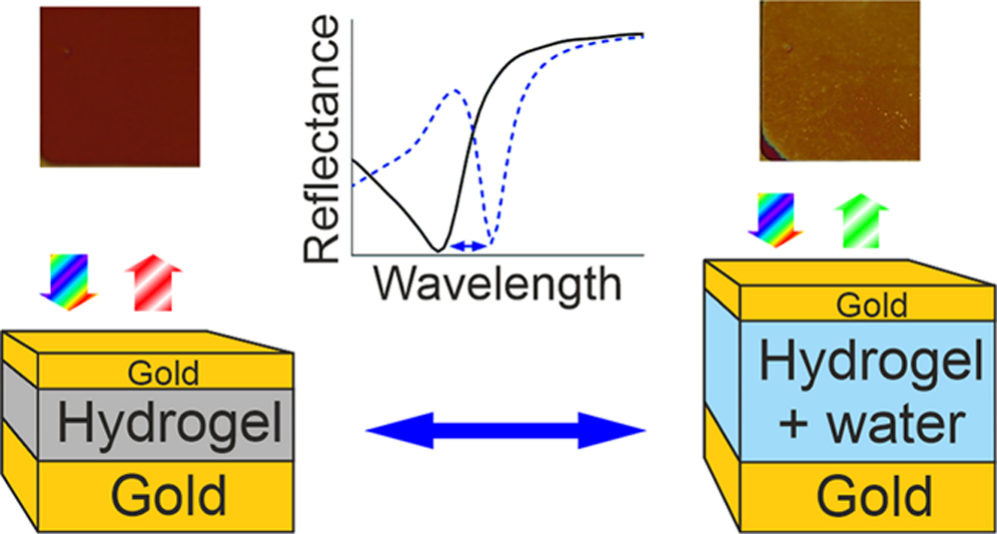

A tunable reflectance filter based on a metal–hydrogel–metal structure responsive to humidity and temperature is reported. The filter employs a poly(*N*-isopropylacrylamide)–acrylamidobenzophenone (PNIPAm–BP) hydrogel as an insulator layer in the metal–insulator–metal (MIM) assembly. The optical resonance of the structure is tunable by water immersion across the visible and near-infrared range. Swelling/deswelling and the volume phase transition of the hydrogel allow continuous reversible humidity- and/or temperature-induced tuning of the optical resonance. This work paves the way toward low-cost large-area fabrication of actively tunable reversible photonic devices.

## 1 Introduction

Since their emergence, plasmonic nanomaterials have been among the fastest-growing subfields of optical materials.^1−3^ Resonant optical absorption and support of highly localized strong electromagnetic fields have yielded many applications for these materials, such as active and passive optical elements, sensors, or energy conversion to mention but a few examples.^4,5^ One direction owing to the opportunity of tailoring the optical absorption^6^ is the development of various types of optical filters.^4,7^ For instance, customizing structural properties of plasmonic nanomaterials to design their spectral response has enabled high-quality color filters.^8^ However, in many cases, this has come with the cost of necessary top-down nanopatterning. To avoid that, lithography-free nanostructures have been developed, relying on thin film deposition and/or bottom-up self-assembly.^9−11^ Prominent examples of lithography-free nanostructures are metal–insulator–metal (MIM) structures^12^ capable of resonant absorption of light and hence sometimes coined as “perfect absorbers”.^13^ The wavelength of the resonance is directly related to insulator thickness in this sandwich structure, as well as to its dielectric properties. Therefore, by changing this thickness, the resonance can be tuned across a wide spectral range.

Lately, there have been many advances in employing MIM structures as color filters in reflection or transmission,^14−16^ as well as studies of other MIM-based applications.^17−19^ There has been interest in making MIM structures actively tunable, widening their applicability in sensorics, optical filtering, and integrated optical devices.^20^ An electrical tuning mechanism was suggested using MIM with an electro-optical insulating layer.^21^ Another approach is using stimuli-responsive insulator layers, which would change the thickness or refractive index and therefore shift the resonance of the MIM structure.^22,23^ One example of such materials is hydrogels, which are responsive to several stimuli.^24−26^ First, they swell remarkably in the presence of water and are responsive to humidity change, which increases the hydrogel’s volume. Second, some hydrogels exhibit a reversible volume phase transition at a lower critical solution temperature (LCST) of the monomers cross-linked into the gel.^27^ For gels, this temperature is also known as volume phase-transition temperature (VPTT). This phase transition results in changing hydrogels from hydrophilic to hydrophobic above the transition temperature, which leads to the expulsion of previously absorbed water and the respective reduction in the hydrogel’s volume. These two volume-tuning mechanisms in hydrogels work oppositely—increasing humidity causes the hydrogel to absorb water and swell, while increasing temperature causes it to expel water and contract.^28^ In the last decade, these properties have brought hydrogels into the field of tunable plasmonic materials,^29−34^ structural coloration,^35−37^ and other optical elements.^38−43^ In particular, Serpe *et al.* have worked extensively on tunable etalons where the dielectric layer comprises PNIPAm microgel, which in aqueous environment can rapidly respond to several stimuli, displaying a color-tuning range of hundreds of nanometers.^33,44−49^ Recently, Jang et al. have demonstrated a humidity-tunable optical transmittance filter for visible range based on an MIM structure with a chitosan hydrogel insulator layer at room temperature,^50^ while Dong et al. have demonstrated a humidity-tunable reflectance filter using cellulose hydrogel.^51^ In both cases, only humidity-induced tuning was studied. To the best of our knowledge, no results have been reported so far combining both humidity and temperature stimuli on hydrogel-enabled mechanisms for optical tuning in air.

Here, we report a bistimuli-tunable hydrogel-based MIM reflection filter. We employ a poly(*N*-isopropylacrylamide)–acrylamidobenzophenone (PNIPAm–BP) hydrogel as the insulator layer, which reversibly changes dimensions under temperature and/or humidity control. The PNIPAm-based hydrogel has a good swelling ratio and good film-forming properties even in the desired 100 nm range.^52^ By applying temperature and humidity stimuli to the PNIPAm hydrogel incorporated into the MIM structure, we obtained continuous spectral tuning. The spectral tuning range amounted to 80 and 340 nm in the visible and near-infrared spectral range, respectively, depending on the initial thickness of the hydrogel. The proposed structures employ low-cost thin film deposition methods such as spin-coating and evaporation, which allows large-area devices and easy scalability. These simple-to-fabricate filters provide actively tunable reversible photonic devices, opening up a range of applications from nano- to macroscale.

## 2 Results and Discussion

### 2.1 Filter Design

**Figure 1:**
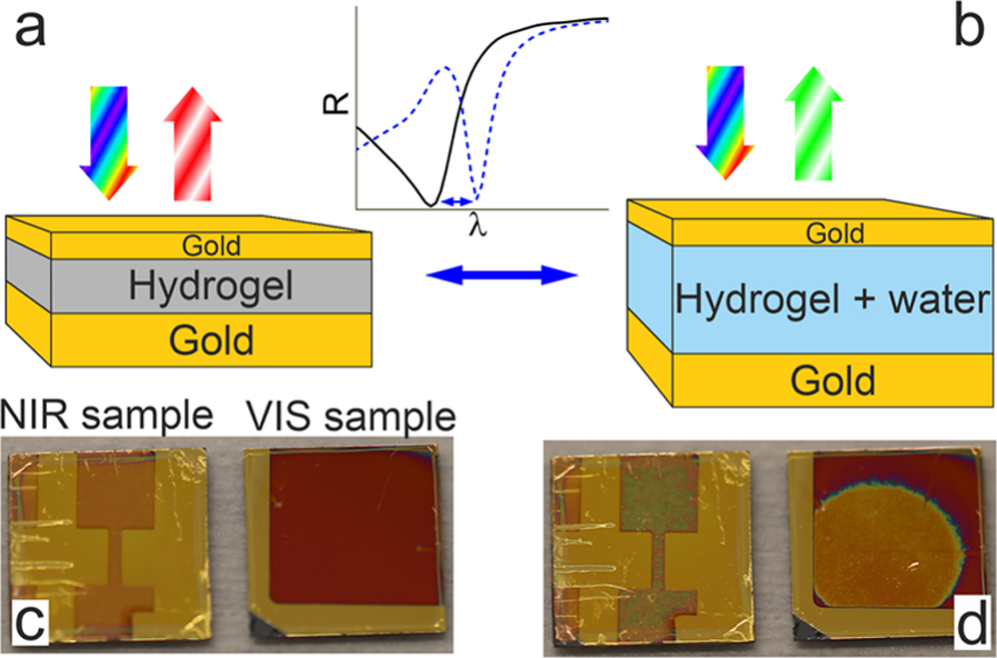
Top: schematic of the hydrogel MIM reflective filter in (a) dry and (b) wet states. The inset graph illustrates the corresponding changes in the reflectance spectra between these two states. Bottom: images of the NIR and VIS samples in (c) dry and (d) wet states.

The MIM structures were fabricated from gold and PNIPAm–BP hydrogel (Figure 1). Gold was chosen for claddings as the nonoxidizing plasmonic layer. The thickness of the bottom layer was set to 100 nm to ensure high reflectivity, while for the top layer, a thickness of 30 nm was used to allow the collection of the reflected light. In this design, the resonant absorption characteristic of the MIM structure translates into the dip in the reflection spectrum; thus, we will address this resonance as reflectance in this paper. Two sets of samples were fabricated, differing in the hydrogel layer thicknesses (80–100 and 200–300 nm). These hydrogel thicknesses correspond to the first-order resonance in the visible (VIS) and near-infrared (NIR) range, respectively, and will be hereon addressed as VIS and NIR samples.

The concept of the hydrogel-based tunable MIM reflective filters is schematically illustrated in Figure 1. The dry state, when hydrogel has no water absorbed, corresponds to a thinner hydrogel layer (Figure 1a). When the hydrogel absorbs water (the wet state), it swells and the resultant thickness increases, thus shifting the resonant reflection band of the MIM structure to longer wavelengths (Figure 1b). Figure 1c,d shows how the observable colors of the samples change between these two states, and the inset in Figure 1 illustrates the corresponding change in the spectra.

### 2.2 Modeling

**Figure 2:**
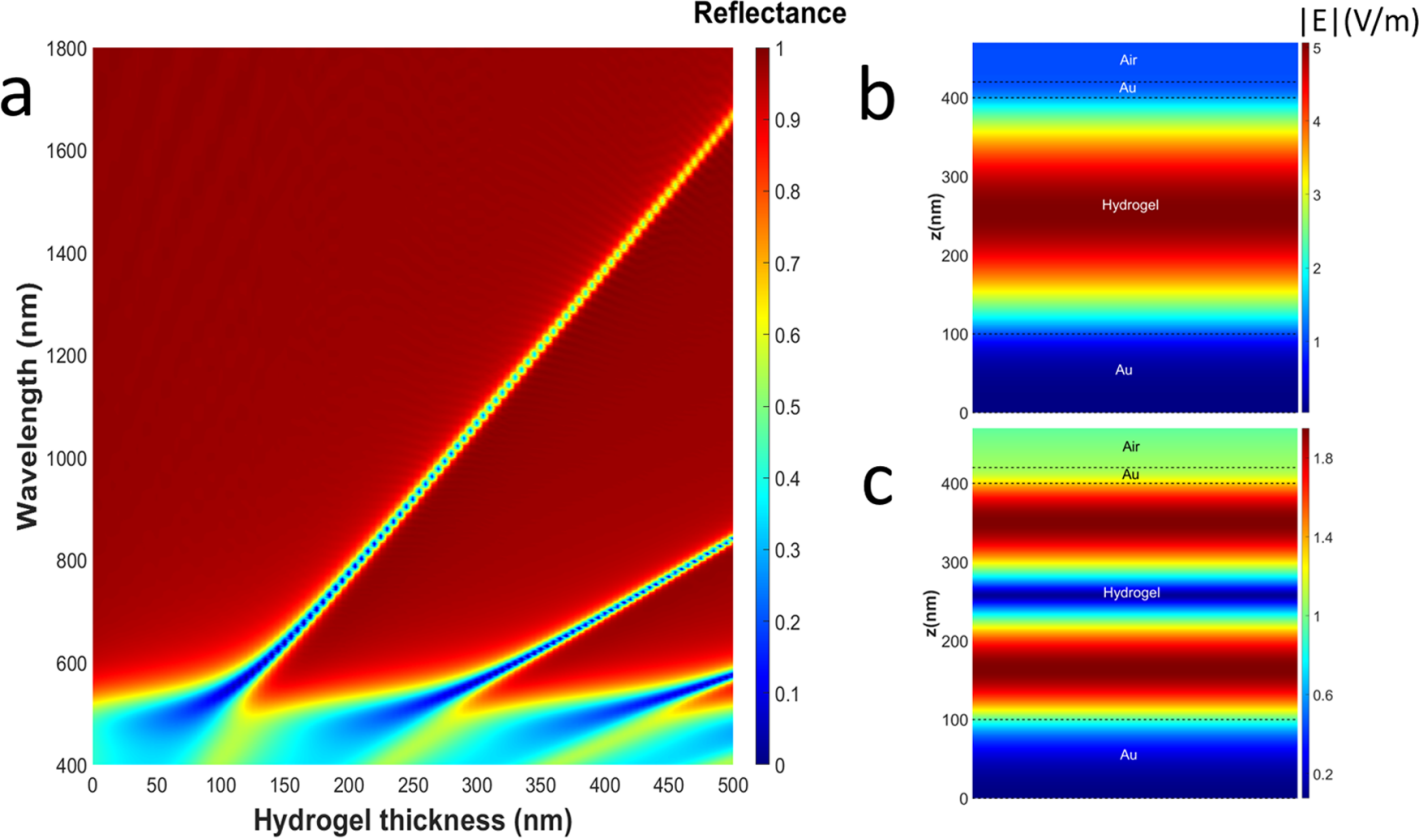
(a) Calculated reflectance of the MIM structure for different thicknesses of the hydrogel insulator layer. Note the presence and tunability of several resonant orders. (b, c) Electric field amplitudes for an MIM with a 300 nm thick hydrogel layer at 1082 nm (first order) (b) and 568 nm (second order) (c).

To design MIM filters and predict their optical properties, we performed finite-difference time-domain (FDTD) calculations of the structures with different hydrogel layer thicknesses. The corresponding reflectance map is shown in Figure 2a. This MIM design allows tunability over a wide range of wavelengths, from just above the gold interband absorption up to at least 1.5 μm. For applications, it is important to consider the possible tuning range of the fabricated MIM structures, which in our case is limited by the swelling factor of the hydrogel (the ratio between wet and dry thicknesses). Assuming the swelling factor to be up to 2,^52^ we designed our VIS samples to have a dry thickness of around 100 nm and NIR one around 250 nm, which should correspond to the maximum achievable tuning range for the first-order resonance of about 550–700 and 900–1600 nm, respectively. It is worth noting that increasing the water content changes the refractive index of the insulator layer toward that of water. However, this difference is less than 0.1 (assuming a 1:1 water:hydrogel mixture for the maximum swelling with a factor of 2)^53^ and therefore is not as influential as the thickness change, though it should slightly reduce the overall tuning range. Importantly, the thicker hydrogel layer also gives rise to higher-order resonances; e.g., in Figure 2a when the thickness exceeds 250 nm, the second order appears, and over 400–450 nm thickness also the third order, and so on. Comparing the electric field distributions for a 300 nm thick hydrogel (corresponding to the partly swollen NIR sample) at resonant wavelengths 1082 and 568 nm (Figure 2b,c, respectively), one can conclude that the latter resonance is indeed the second order. These higher-order resonances are also tunable with the thickness change, though with less sensitivity compared to the first-order resonance. However, this lesser sensitivity is compensated by the fact that a higher initial hydrogel thickness yields a higher absolute thickness change at the same swelling ratios.

### 2.3 Tuning by Water Immersion

**Figure 3:**
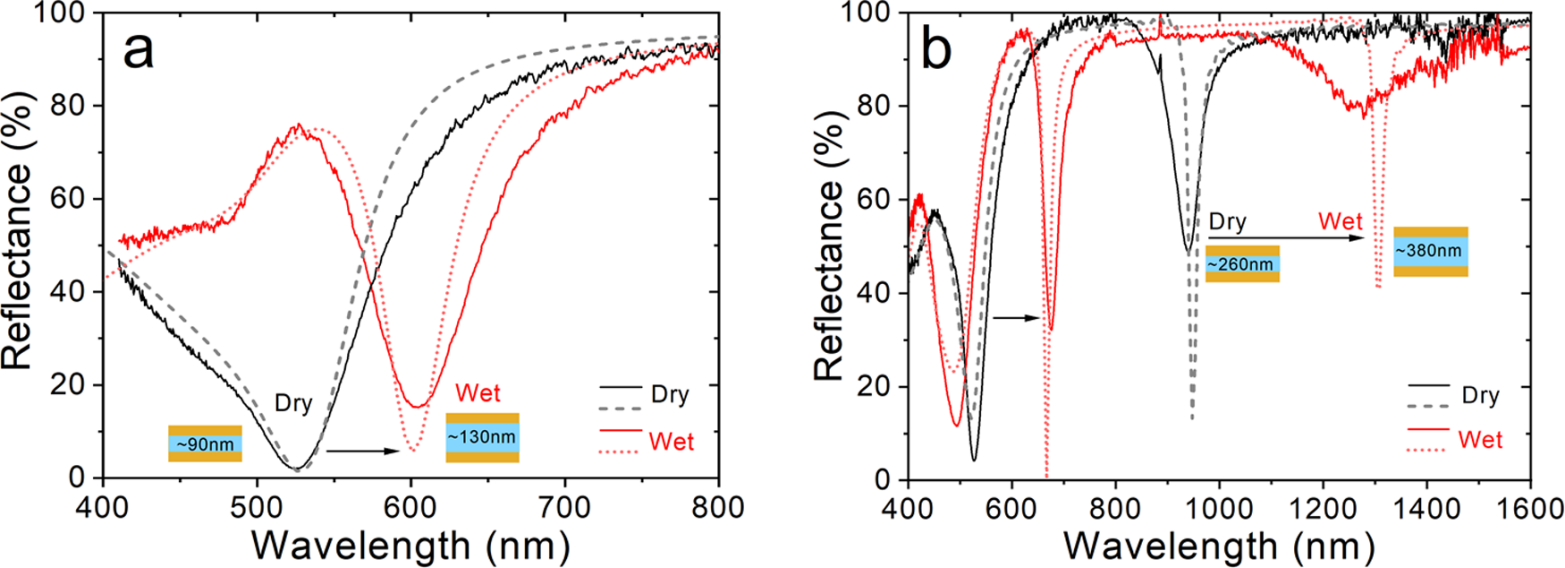
Reflectance of hydrogel-based MIM filters at ambient conditions and after immersion in water. (a) VIS sample and (b) NIR sample. Solid, experimental spectra; dashed and dotted, modeling. The arrows show the shift of the resonant dips.

To evaluate the maximum tuning range of the MIM structures, we compared their reflectance spectra at ambient conditions (26 °C, 30% relative humidity) and after immersion in deionized water for 20 min (Figure 3). The dry samples demonstrate reflectance dips almost to 0%, characteristic of MIM structures. The reflectance dips move to longer wavelengths after the samples are immersed in water; the corresponding color changes are evident in Videos S1 and S2. The penetration of water into the hydrogel layer is supposedly enabled by the porosity of the sputtered gold film,^54^ and local variations of this porosity together with spin-coating defects (Figure S4) explain the observed inhomogeneities of swelling. The wavelength of the resonant absorbance of the MIM structure depends on the thickness of the insulator; therefore, swelling of the hydrogel increases the resonant wavelength of the filter.

The experimental results were fitted to the modeling presented in Figure 2. The validity of the model was confirmed by the perfect match between the reflectance spectra calculated for MIM structures with the hydrogel thicknesses of 90 and 260 nm and the experimental spectra for VIS and NIR samples with the measured hydrogel thicknesses of 85 ± 5 and 250 ± 10 nm, respectively. The observed broadening of the experimental resonances in comparison to the modeling, as well as the experimental data reported for solid-state MIMs elsewhere,^15^ can be explained by fabrication imperfections, originating mostly from spin-coating. Fitting the same model with different hydrogel layer thicknesses to the wet experimental spectra allowed us to estimate the thickness change. We considered a constant refractive index of 1.503 for the hydrogel layer in all cases; therefore, the fitted thicknesses in the wet case are slightly underestimated.

The estimated change in the hydrogel thickness was from 90 to 130 nm for the VIS sample and from 260 to 380 nm for the NIR sample, which corresponds to a 1.4–1.5 swelling factor. The respective overall spectral shift of the resonance was 80 nm for the VIS sample (from 524 to 604 nm) and 340 nm for the NIR sample (from 940 to 1280 nm). In addition to that, the second-order resonance of the NIR sample shifted by 150 nm (from 524 to 674 nm). The latter illustrates that thicker hydrogel layers may be more beneficial for applications, as higher initial thickness allows a broader tuning range at a constant swelling ratio, while higher-order resonances give access to the visible wavelength range. Importantly, the swelling did not reduce the quality factors of the resonances: *Q*-factors (*Q* = λ/Δλ; Table S1) exceeded 10 in a 650–1000 nm wavelength range for both dry and wet states, and the maximum *Q*-factor of around 19 was measured for the second-order resonance in the wet state (dip at 675 nm in Figure 3b; FWHM = 35 nm). This is in line with the modeling predictions for the highest *Q*-factors in this wavelength region, with no regard to water presence, so it is solely defined by the MIM geometry. This value is reported for the first time for environment-responsive MIM structures, and it is close to *Q*-factors reported for traditional solid-state MIM structures^3,14,18^ and can supposedly be further improved with better uniformity of thin layer deposition.

### 2.4 Tuning by Temperature and Humidity Control

**Figure 4:**
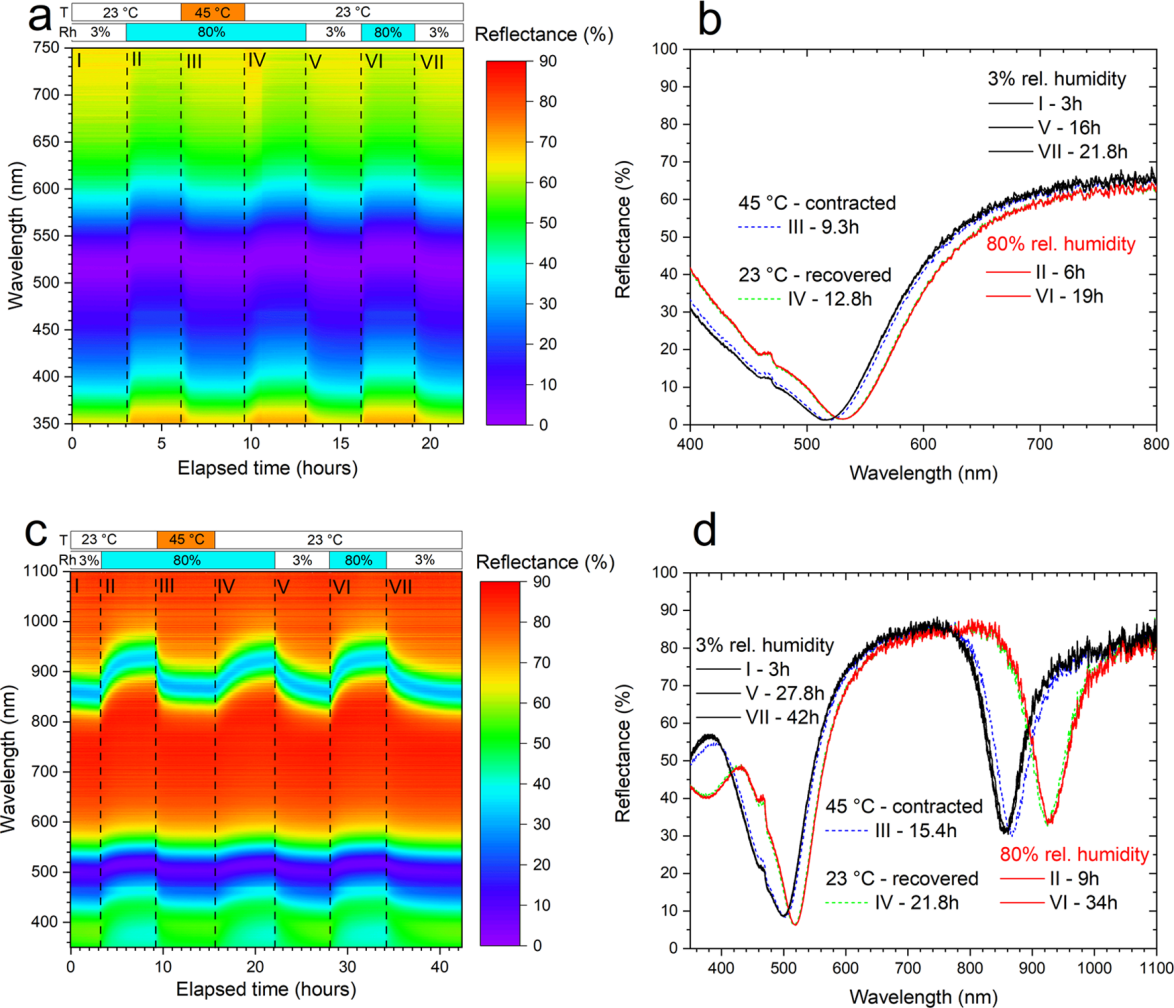
Reflectance spectra at varied environmental conditions: (a, b) VIS sample and (c, d) NIR sample. (a, c) Time dependencies of the reflectance spectra; time steps II–VI are 3 h (a) and 6 h (c). The sample temperature and relative humidity of the environment at different times are shown in the bars on top of the graph. (b, d) Reflectance spectra at the end of each time step.

We employed temperature- and humidity-controlled chambers to demonstrate continuous and reversible tuning of the optical resonance in the hydrogel MIM filter in air. Continuous measurements of the reflectance spectra in a controlled environment showed that both humidity and temperature can be used for tuning the reflectance of the fabricated structures (Figure 4). This employs two mechanisms. An increase in humidity at constant temperature results in hydrogel swelling, which corresponds to the red shift of the resonance of the MIM structure (transition from I to II in Figure 4a,c), and vice versa—decreasing humidity “dries” the hydrogel and blue-shifts the resonance (Figure 4a,c, V). Another mechanism specific to PNIPAm-based hydrogels is to utilize the LCST phase transition at a temperature around 32 °C,^55^ above which the swollen hydrogel contracts. Importantly, it is a reversible transition. To use this mechanism, we changed the temperature of the MIM structure while keeping a constant humidity of 80% (Figure 4a,c, III and IV). At room temperature, the hydrogel is partly swollen at this humidity (Figure 4a,c, II). However, increasing the temperature up to 45 °C resulted in the blue shift of the resonance to the values characteristic to the MIM with dry hydrogel (Figure 4a,c, III), meaning that the phase transition took place and the hydrogel contracted, expelling water from it. The close spectral match between the temperature-contracted (Figure 4a,c, III) and dry hydrogels (at 3% relative humidity; Figure 4a,c, I) indicates that the adsorbed water is almost completely gone at 45 °C. It is worth noting that this transition is faster than the humidity-driven swelling/deswelling (compare the slope in Figure 4c, III and V). We also demonstrated the reverse effect; i.e., lowering the temperature (while keeping 80% humidity) moved the resonance back to the longer wavelengths (Figure 4a,c, IV). One more cycle of humidifying-drying was added to show reproducibility of the hydrogel swelling/deswelling (Figure 4a,c, VI and VII). The spectra at the end of each step I–VII in Figure 4a,c are shown in Figure 4b,d, respectively, demonstrating perfect reproducibility of the tuning of the resonance during subsequent swelling–deswelling cycles. The overall spectral tuning range was up to 80/70 nm in the near-infrared and 22/18 nm in the visible region for humidity/temperature-induced mechanisms. These values were both achieved with the NIR sample (the first- and the second-order resonances, respectively); for the VIS sample, the tuning range was 15/12 nm. This supports the hypothesis that employing the higher-order resonances in the visible range using a thicker initial hydrogel layer may be beneficial for the maximal tuning range.

**Figure 5:**
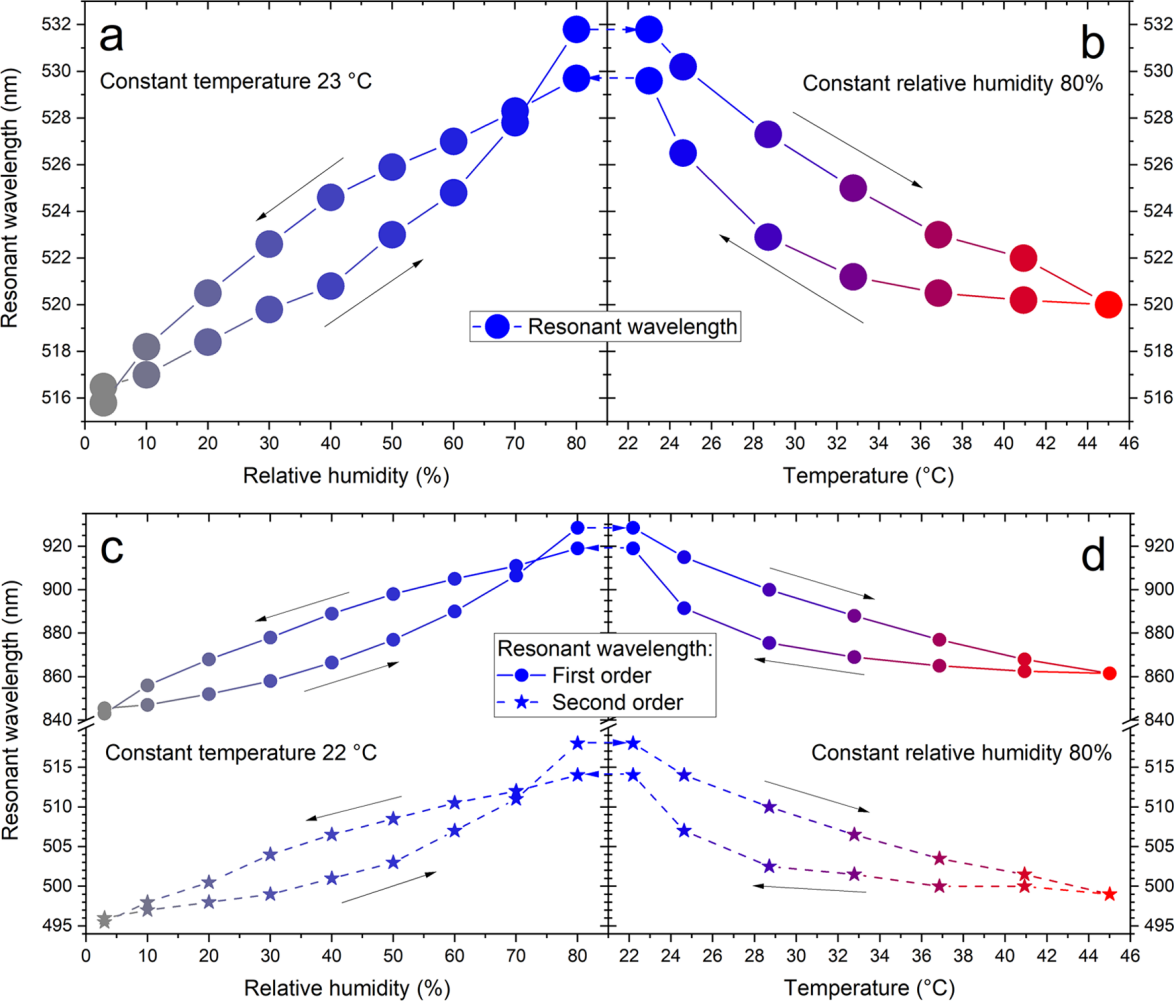
Dependency of the resonant wavelengths on (a, c) humidity (at ambient temperature) and (b, d) temperature (at 80% rel. humidity) for (a, b) VIS and (c, d) NIR samples. Note the different vertical scales in panels (c, d) before and after the break. Lines are for eye-guiding only. Raw measurements (spectra, humidity, and temperature) are available in SI, Figures S7 and S8.

Figure 5 shows the dependencies of the resonance in the samples when temperature and humidity are gradually changed between the same extreme values (3–80% RH, 23–45 °C, the system was let to stabilize at each value for 3 h). A full spectroscopic map for this measurement is available in the Supporting Information. Gradual changes in humidity expectedly allow fine-tuning of the resonance with close to linear dependency (0.19 nm/% RH) with almost no hysteresis (Figure 5a,c). At the same time, the temperature-induced changes at high humidity are also continuous, not step-like as one could expect from a phase-transition-induced process, allowing continuous tuning as well (Figure 5b,d).

While PNIPAm-based hydrogels are generally known to undergo rapid LCST transition within a few °C range, this range, as well as the transition temperature, can be tuned by additives (e.g., comonomers).^56^ In our experiments, we employed a benzophenone (BP) comonomer, the hydrophobicity of which is expected to lower the LCST. It also provides more linear temperature changes to the PNIPAm hydrogel.^57,58^ However, our hydrogel constitutes from 48:1 composition of PNIPAm:BP (further details in SI), so the influence of the comonomer is supposedly not strong enough to extend the transition range to a couple of tens of °C (in^57,58^ this ratio was in the range of 12:1). Additionally, the transition temperature is traditionally estimated for hydrogel in water, while it depends on the water content in the hydrogel,^59^ which was significantly lower in our humidity-driven experiment. Finally, the temperature-tuning of the PNIPAm hydrogel volume above LCST has been reported;^60^ however, the shrinking was irreversible, which differs from our samples.

We believe that the temperature hysteresis observed in Figure 5b,d can be explained by the formation of a dense skin layer at the beginning of the deswelling process, as reported elsewhere.^61^ To date, the temperature-induced volume changes in PNIPAm hydrogels have been mostly studied in water,^62,63^ while the mechanism of the observed changes in humid air across a wide range of temperatures is yet to be understood and it can broaden possible PNIPAm hydrogel applications beyond temperature-tunable color filters reported here.

## 3 Conclusions

We report a continuously tunable reflective color filter based on the metal–insulator–metal (MIM) structure with a hydrogel as an intermediate insulating layer. The device responds to two stimuli—humidity and temperature—by reversibly changing the resonant wavelength. We obtained a spectral red shift of 22 nm in the visible range and 80 nm in the near-infrared range by changing the humidity from 3 to 80% for the initial hydrogel thickness at ambient conditions of 90 and 260 nm, respectively. The maximum resonance shift obtained by immersion of the hydrogel MIMs in water was 80 and 340 nm, respectively. These shifts are attributed to the reversible swelling of hydrogel in the presence of water and deswelling when water is removed. The temperature-tuning mechanism employed the LCST phase transition when swollen (wet) hydrogel contracts at elevated temperatures expelling water from it. We show that changing the temperature from ambient to 45 °C at a constant 80% humidity results in the continuous blue shift of the resonant wavelength with the maximum shift of 18 nm in the visible range and 70 nm in the near-infrared, and these changes are reversible. We also demonstrate that for better tunability it is beneficial to employ the higher-order resonances, as in our experiments the second-order resonance outperformed the first-order one in the overall tuning range and thus in humidity and temperature sensitivity. The best experimental quality factor of around 19 was obtained in the visible range for the second-order resonance in MIM with the swollen hydrogel, indicating that swelling does not reduce the quality of the resonances. The reported tunable filter opens new perspectives for plasmonic devices such as contactless (optical) sensors of humidity or temperature of the liquid. Another possible direction is using the proposed structure as a tunable cavity for emitters embedded in the hydrogel.

While our samples demonstrated good resonant properties, it is possible to further improve the optical properties by improving the deposition quality. We believe that further extension of the tuning range is possible using higher-refractive-index liquids in addition to water, the aqueous mixtures of which can be compatible with bistimuli tuning in a certain range of concentrations. An important advantage of the lithography-free MIM structures is that they do not require nano- or micropatterning, relying only on thin film deposition methods, which makes these structures potentially scalable for roll-to-roll industrial manufacturing.^64,65^ The demonstrated stimuli-responsive structures can be applied as humidity sensors or immersed temperature sensors—in both cases, sensing would be optical and therefore contactless. Leaning on plasmon resonances, MIMs can also be used as Raman-enhancing substrates,^66,67^ which in combination with temperature/humidity sensing opportunities gives rise to potential integrated applications, e.g., in lab-on-chip.

## 4 Methods

### 4.1 Numerical Calculations

The reflectance spectra of the metal–hydrogel–metal thin film, as well as E-field distributions under the plane-wave irradiation, were simulated using the two-dimensional (2D) finite-difference time-domain (FDTD) method with commercial software (Lumerical FDTD Solutions). The modeled structure comprised five layers (from bottom to top): (1) semi-infinite glass substrate, (2) 100 nm thick gold, (3) hydrogel of a varied thickness (0–500 nm), (4) 30 nm thick gold, and (5) semi-infinite free-space superstrate. The refractive indices of the glass, the hydrogel, and the superstrate were taken as 1.46, 1.503 (see Supporting Information, Figure S10), and 1, respectively, while the complex index of gold was in accordance with Johnson and Christy data.^68^ The plane-wave excitation was incident from the superstrate perpendicular to the layers (along the x-direction). A power monitor was placed at a distance of half the maximum wavelength behind the radiation source to measure the reflectance spectra. The mesh refinement was set to the conformal variant 0 with a minimum mesh set of 0.25 nm. Perfectly matched layer (PML) boundary conditions were set along the *x*-direction, and the antisymmetric boundary condition was set along the *y-*direction. The number of layers was increased to prevent any divergence in the simulation due to the dispersive Au unit cell. The auto-shutoff min was set to 1e-6, which was enough for the incident field to decay completely, as well as prevent any ripples in the reflectance spectra. The attained simulated results were used to fit the experimentally measured reflectance spectra of the samples in both dry and wet cases.

### 4.2 Hydrogel Material Synthesis

The *N*-isopropylacrylamide–acrylamidobenzophenone copolymer (PNIPAm–BP) was synthesized from commercial *N*-isopropylacrylamide and freshly synthesized 4-acrylamidobenzophenone via free-radical polymerization initiated by azobisisobutyronitrile (AIBN). The composition of the polymer was confirmed by ^1^H NMR spectroscopy to be (NIPAm_48_BP_1_)_*n*_, well in line with the monomer ratio before polymerization. Details of the synthetic protocols and characterization of the synthesis products are given in Supporting Information, Figures S1 and S3.

### 4.3 Hydrogel Layer Fabrication

PNIPAm–BP copolymer was diluted in 94% ethanol in concentrations 20 and 40 mg/mL; for better dissolution, the magnetic stirring at 50 °C was used for 1 h. Before spin-coating, the solutions were filtered through PTFE membranes with 0.45 μm pores. The substrates were cleaned by consecutive sonication in acetone, isopropanol, and deionized water (10 min each). After that, they were activated by plasma treatment (20 min, 30 W RF power, 1000 mTorr O_2_) and immediately forwarded to spin-coating. Spin-coating included two steps: (1) 10 s at 150 rpm during which the PNIPAm–BP solution was dispensed and predistributed and (2) 30 s at 1000–6000 rpm to form the final coating. The deposition was followed by drying for 1 h at 40 °C in a vacuum and cross-linking under UV light (365 nm from CoolLED pE-4000 focused into a circle of ca. 2 cm in diameter) for 40 min. Complete cross-linking at these conditions was confirmed by the disappearance of the 301 nm peak in the optical transmittance spectra of the reference hydrogel coatings on glass (see the Supporting Information for more information).

### 4.4 MIM Structure Fabrication

The samples were fabricated on Si wafer square pieces. After consecutive sonication in acetone, isopropanol, and deionized water (10 min each), the substrates were blow-dried with nitrogen. After that, the adhesive layer of 1 nm Ti followed by a 100 nm layer of Au was deposited by e-beam evaporation. The hydrogel layer was fabricated as described before. The final gold layer (20–30 nm) was made by thermal sputtering. The thicknesses of all gold and hydrogel depositions were verified at the reference samples with the Dektak profilometer at cleanroom conditions (21 °C, 20–25% relative humidity). The resulting thicknesses were 100 nm Au/85 ± 5 nm hydrogel/30 nm Au for VIS samples and 100 nm Au/250 ± 10 nm hydrogel/26 nm Au for NIR samples.

### 4.5 Optical Measurements

Microscopic reflectance measurements were performed with a multifunctional WITec alpha300C confocal microscope. The samples were illuminated by a broad-band light source (LDLS EQ-99X) through a Zeiss EC “Epiplan” DIC, 20× air objective (NA = 0.4, WD = 3.0 mm); the reflected light was collected through the same objective and coupled to spectrometers via an optical fiber. For the spectral range of 400–800 nm, we used a WITec UHTS300 spectrometer equipped with 150 lines/mm grating and a TE-cooled CCD camera (Andor DV 401-BV-351). For the NIR spectral range (800–1600 nm), an Ocean Insight NIRQUEST 512-XR FLAME spectrometer equipped with an InGaAs linear array detector was used. Samples were measured at room conditions (26 °C, 30% r.h., dry state) and after immersion in deionized water for 1 h (wet state). An extended set of experimental spectra fitted with modeling results are shown in Figures S5 and S6.

Measurements in a controlled temperature and humidity environment were performed using the Linkam Scientific LTS420-H stage with an RH95 humidity controller, providing precise humidifying/dehumidifying of the air in the sample chamber, and a T96-S temperature controller for heating the sample. The temperature of the sample was verified using an IR camera (Figure S9). Optical reflectance, in this case, was measured with the help of a Thorlabs RP29 reflectance fiber probe, which was connected to both an Ocean Optics DH-2000-BAL light source (halogen and deuterium lamps) and Avantes AvaSpec-2048L fiber spectrometer. The light to/from the fiber was coupled with a Thorlabs RC08SMA-F01 reflective collimator and weakly focused with Thorlabs LA4600 fused silica lens. The illuminated area on the sample was roughly 0.5 mm in diameter.

In all experiments, the reference intensity (reflected light source) and background/dark intensity were measured using a silver mirror with the light sources being, respectively, on and off. The reflectance of the sample was calculated as follows
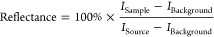
1where *I*_Sample_ is the measured intensity of the light reflected from the sample, *I*_Source_ is the reference light source intensity, and *I*_Background_ is the background intensity.

The absorbance of the reference hydrogel layers on the glass was measured with Agilent Technologies Cary 60 UV–vis spectrophotometer with a custom-built sample chamber.

## Supporting Information Available

Hydrogel material synthesis and characterization; metal–hydrogel–metal samples; spectral measurement in the temperature- and humidity-controlled environment; and refractive index of the PNIPAm hydrogel (PDF)
Video S1 of color changes in VIS and NIR samples when water is being added and removed (MP4)
Microscopic video S2 of color changes in the VIS sample when water is being added and removed (MP4)
