# Supplementary material for: Humidity- and Temperature-Tunable Metal–Hydrogel–Metal Reflective Filters
Source: ACS Appl Mater Interfaces. 2021 Oct 13;13(42):50564–72. doi: 10.1021/acsami.1c15616 (PMC8554756; doi:10.1021/acsami.1c15616)
Supplement: Supplementary file 1 — am1c15616_si_001.pdf [file am1c15616_si_001.pdf]

Supporting Information: Humidity- and Temperature-Tunable Metal-Hydrogel-Metal Reflective Filters

Semyon Chervinskii, Ibrahim Issah, Markus Lahikainen, Alireza R. Rashed, Kim Kuntze, Arri Priimagi,* and Humeyra Caglayan*

Faculty of Engineering and Natural Sciences, Tampere University, 33720 Tampere, Finland

E-mail: arri.priimagi@tuni.fi; humeyra.caglayan@tuni.fi

1 Hydrogel material synthesis and characterization

1.1 Acrylamidobenzophenone synthesis

4-aminobenzophenone (1.0 g, 5.1 mmol) was dissolved in 20 ml of dry dichloromethane under nitrogen. Dry triethylamine (1.08 ml, 8.2 mmol) and acryloyl chloride (0.68 ml, 8.2 mmol) were then added sequentially. The reaction mixture was stirred for 2.5 hours and then quenched with water, washed with water and concentrated NaCl solution, dried with magnesium sulfate, filtered and concentrated under reduced pressure. The crude product was purified by column chromatography (dichloromethane) to yield the product (562 mg, 44%) as a pale yellow solid. ^{1}H NMR (500 MHz, $CDCl_3$): δ 7.83 (d, J = 8.6 Hz, 2H), 7.77 (d, J = 6.9 Hz, 2H), 7.71 (d, J = 8.6 Hz, 2H), 7.58 (t, J = 7.4 Hz, 1H), 7.48 (t, J = 7.7 Hz, 3H), 6.48 (d, J = 16.6 Hz, 1H), 6.27 (dd, J = 17.2, 10.3 Hz, 1H), 5.83 (d, J = 11.5 Hz, 1H) (**Figure S1**).

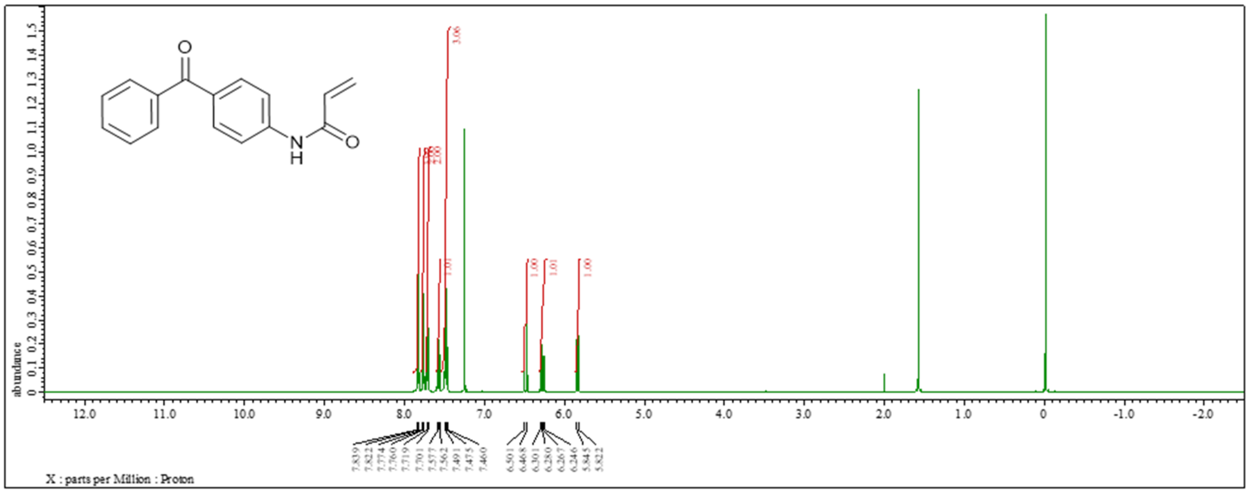

Figure S1: NMR of synthesized acrylamidobenzophenone material.

1.2 PNIPAm-BP copolymer synthesis

Copolymer was synthesized by free-radical polymerization. N-Isopropylacrylamide (NIPAm, 937 mg, 8,3 mmol), acrylamidobenzophenone (BP, 43 mg, 0.17 mmol) and azobisisobutyronitrile (AIBN, 24 mg, 0.15 mmol) were polymerized in 10 ml of 1,4-dioxane at 70 °C for 24 h under nitrogen resulting PNIPAm-BP copolymer. The copolymer was purified by precipitation into stirring diethyl ether and dried overnight in a vacuum oven at room temperature.

1.3 Composition of the copolymer

Composition of the copolymer was determined by measuring an ^{1}H NMR spectrum (in DMSO-d_6) (**Figure S2**). The polymer composition was estimated by comparing the integrals of the methyl peaks of the NIPAm monomer (6 protons per unit) and the aromatic signals of the acrylamidobenzophenone (9 protons per unit). The integral ratio (aromatic:methyl) is 1:32, and when the numbers of protons in each unit are taken into account, the composition is estimated to be 48:1 (NIPAm:BP). This is well in line with the ratio of monomers before polymerization (50:1).

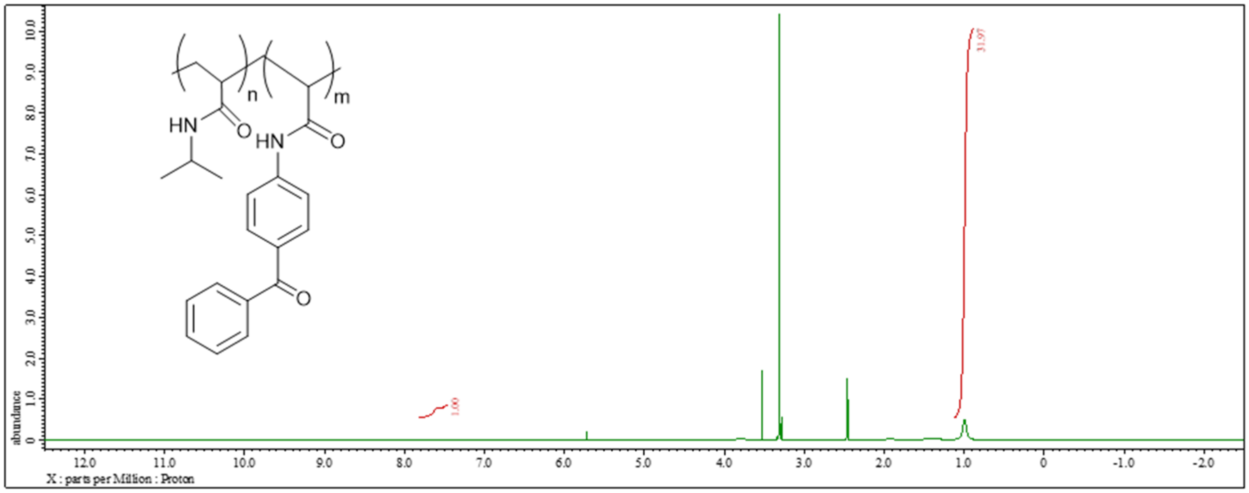

Figure S2: NMR of synthesized PNIPAm-BP copolymer.

1.4 Crosslinking characterization

The kinetics of crosslinking upon UV exposure was measured with UV-Vis spectroscopy. Small amount of PNIPAm-BP ethanol solution (0.2 mg/ml) was sandwiched between two quartz plates and spectra were measured before and after 33 mW/cm^2 of 365 nm UV illumination (**Figure S3**). During the UV illumination, the benzophenone is excited to a singlet state which rapidly undergoes intersystem crossing to a diradical triplet state. The formed radical reacts with a C–H in another polymer chain, forming a C–C bond and crosslinking the polymer (scheme in Figure S3). This will occur within the timespan of ∼10 min (inset of Figure S3).

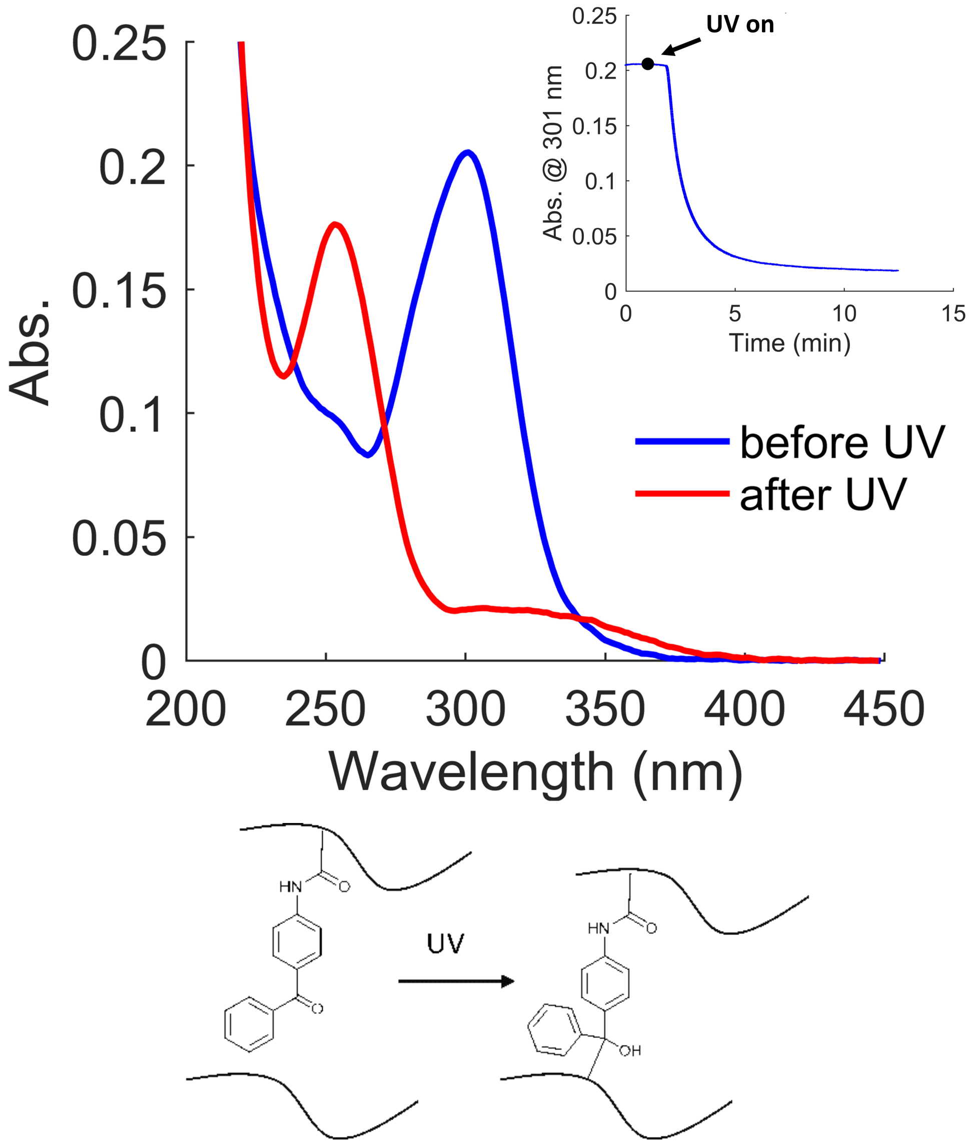

Figure S3: Absorbance spectra of PNIPAm-BP ethanol solution before and after UV irradiation.

2 Metal-hydrogel-metal samples

Several samples were fabricated with varied hydrogel layer thickness. The hydrogel thickness was controlled by a) solution concentration (2 concentrations were used, 20 and 40 mg/ml), and b) spin-coating speed (after first 10 s of predispensing at slow speed, rpm were increased to 1000-6000). Two samples with good uniformity providing the most consistent results (across the sample and between the measurement) were chosen for the main manuscript as VIS and NIR ones. **Figure S4a** shows overview of the VIS sample with the patterned top gold layer, it is possible to see the quality of both gold and hydrogel coatings, and high magnification Figure S4b illustrates the local porosity of thin top gold layer, enabling water penetration. Spectral measurements of all "VIS" samples in wet and dry state are presented in **Figure S5**. **Figure S6** shows and example of the measurements at different points of one of "NIR" samples. **Figure S1** shows the resonant wavelengths and corresponding Q-factors for the spectra presented in Figure 3 in the main text.

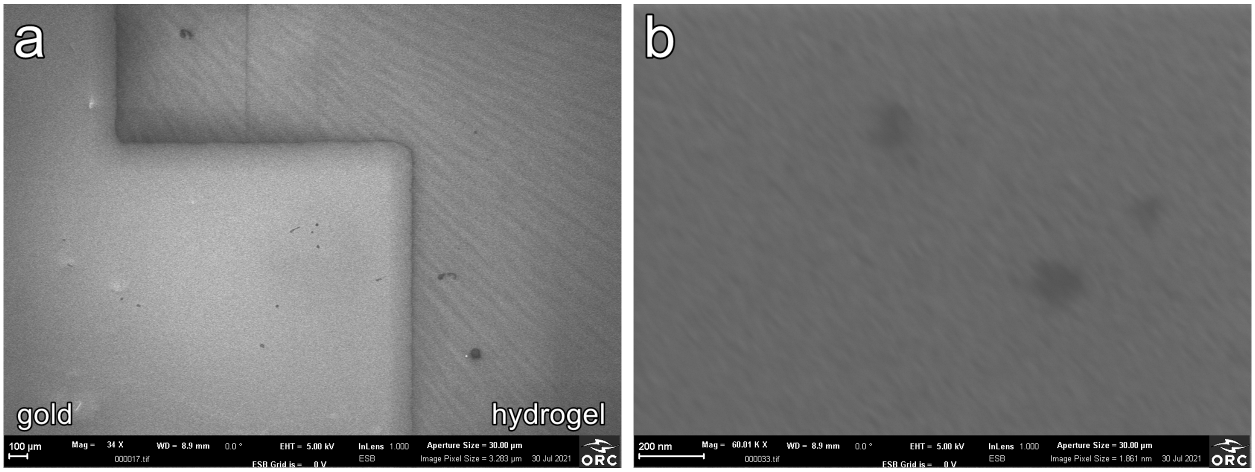

Figure S4: SEM images of VIS sample. a) Overview of the sample. b) Central area in high magnification.

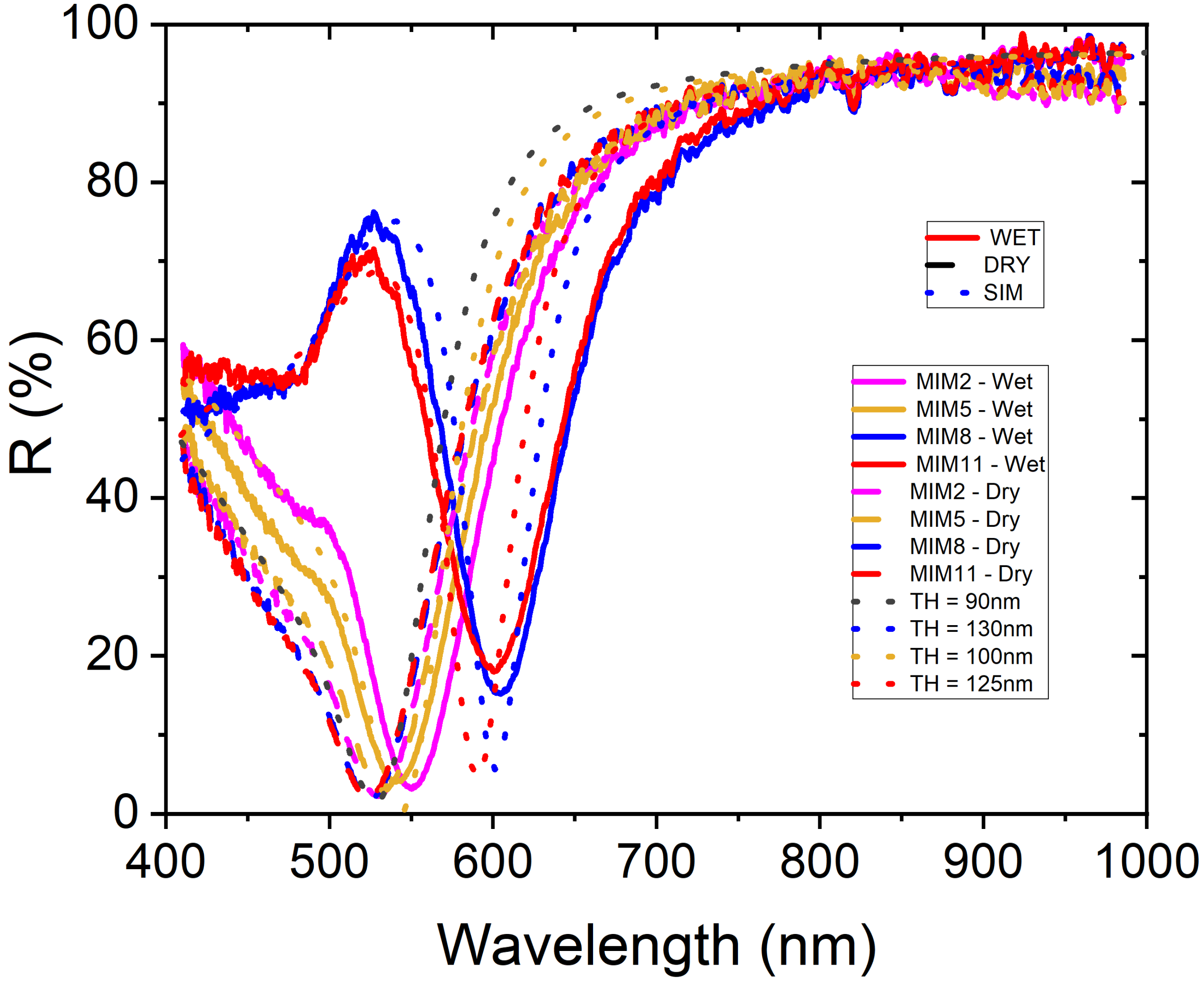

Figure S5: Reflectance spectra different samples with "dry" hydrogel layer thickness of around 90 nm before (dashed) and after (solid) immersion in water. Short dash - modeled spectra for the structures with different thickness of hydrogel layer.

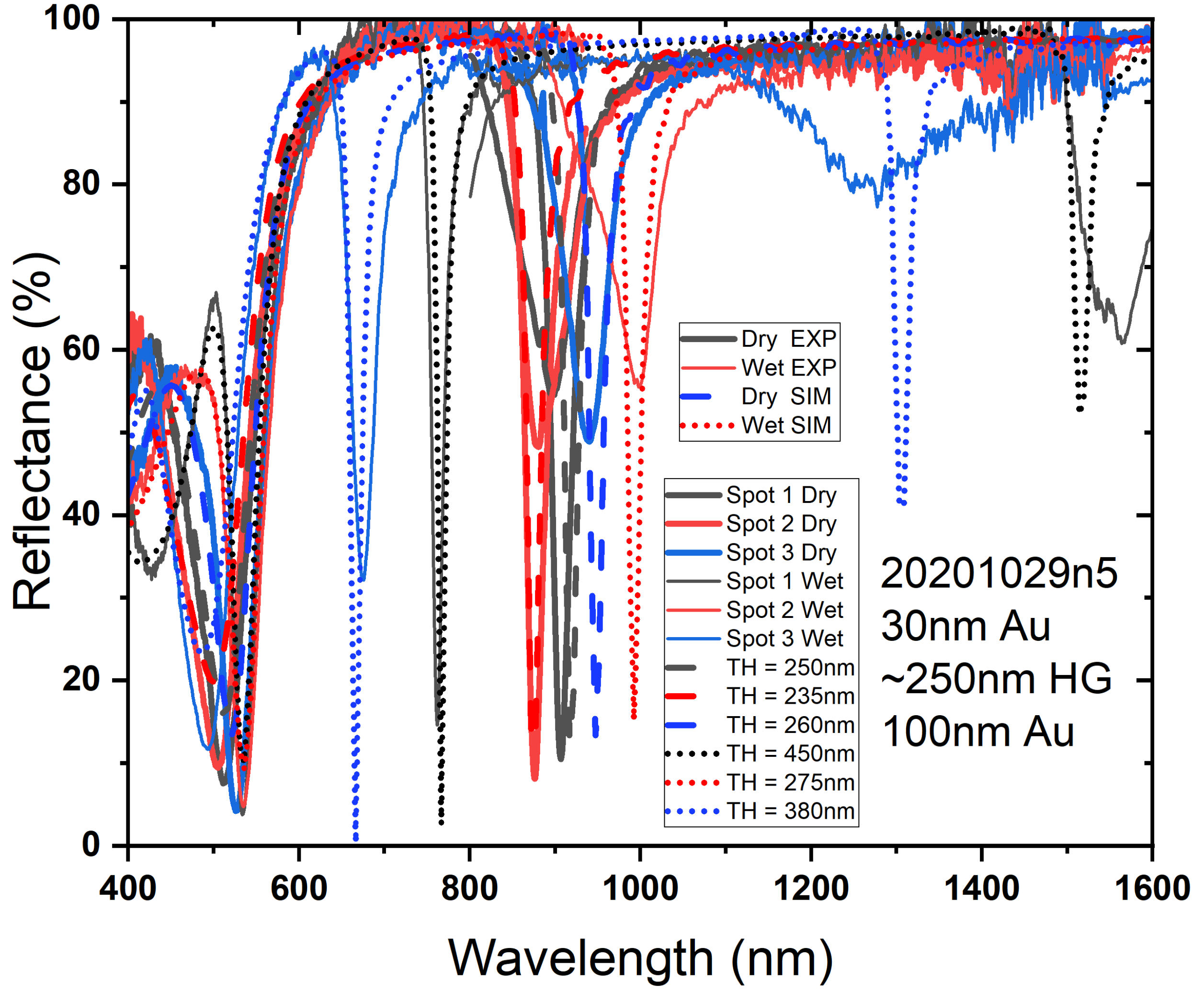

Figure S6: Reflectance spectra at different points of a sample with "dry" hydrogel layer thickness of around 250 nm before and after immersion in water.

Table S1: Resonant wavelengths and corresponding quality factors for VIS and NIR samples, extracted from spectra in Figure 3 in the main manuscript.

Sample	Resonant order	Dry		Wet	
		Resonant wavelength, nm	Q-factor	Resonant wavelength, nm	Q-factor
VIS	1	525	4.3	604	7.6
NIR	1	940	15.2	1275	7.1
	2	527	9.6	675	19.3
	3	n/a	n/a	493	6.3

3 Spectral measurement in the temperature- and humidity-controlled environment

The full spectroscopic maps for the data presented in Figure 5 in the main manuscript is shown in **Figure S7**. **Figure S8** shows the example of the recorded humidity and temperature values (for Figure S7b) to illustrate the stability and precision of the control provided by the experimental setup. **Figure S9** shows the FLIR camera image of the sample and Si wafer on the stage heated to to 50 °C. Using the measurement we assumed that linear change of the stage temperature from ambient to 50 °C corresponds to linear change of the sample temperature from ambient to 45 °C.

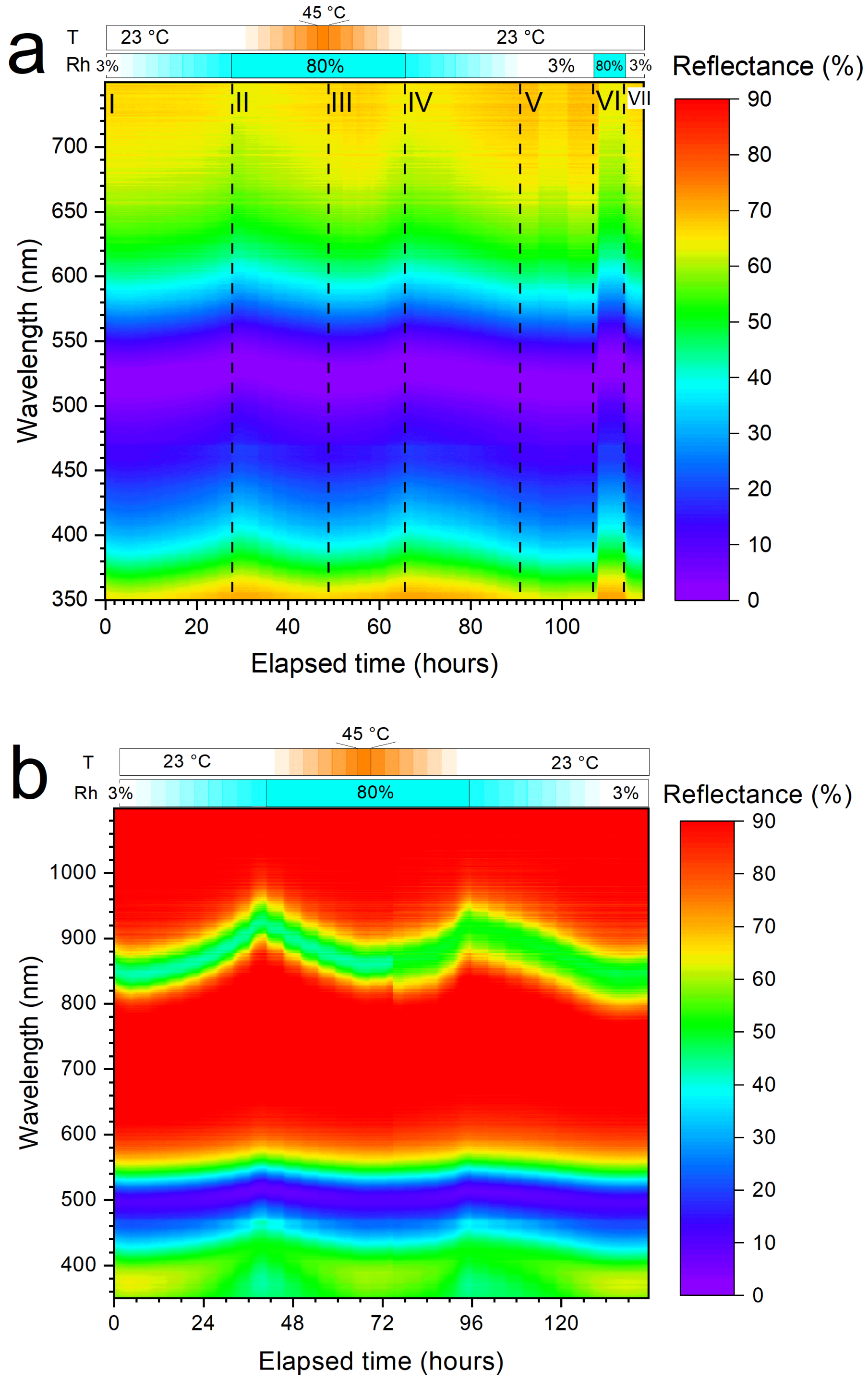

Figure S7: Reflectance spectra of a) VIS and b) NIR samples at gradually changing humidity and temperature. Sample temperature and relative humidity of the environment are shown in the bars on top of the graph.

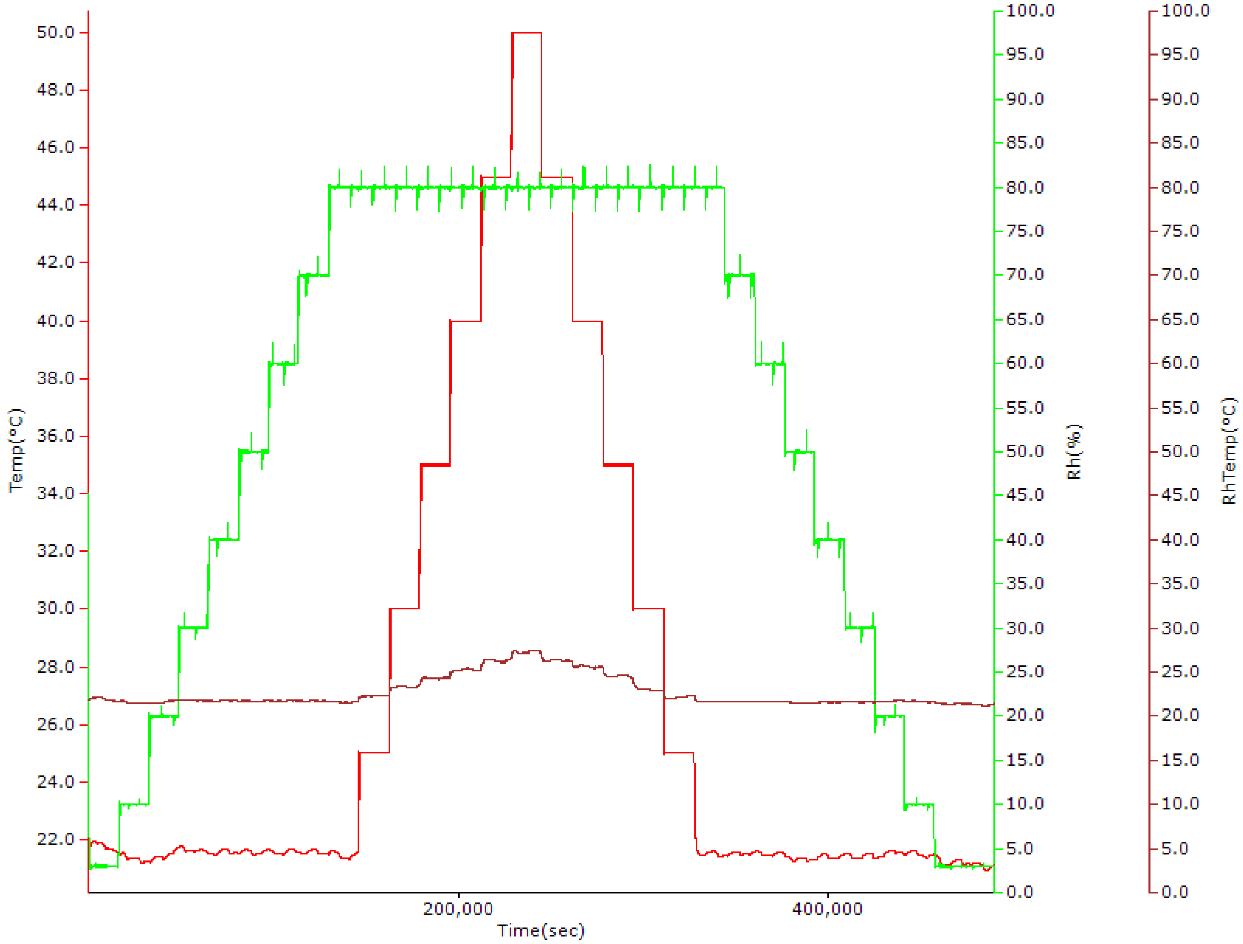

Figure S8: Raw humidity and temperature data for the measurement in Figure S6b, Rh and RhTemp are relative humidity and temperature of the air inside humidity chamber, Temp is the temperature of the heater (under the sample). Note that vertical green line in the left part represents the quick drop in humidity at the beginning of the measurement, not the color of the axis.

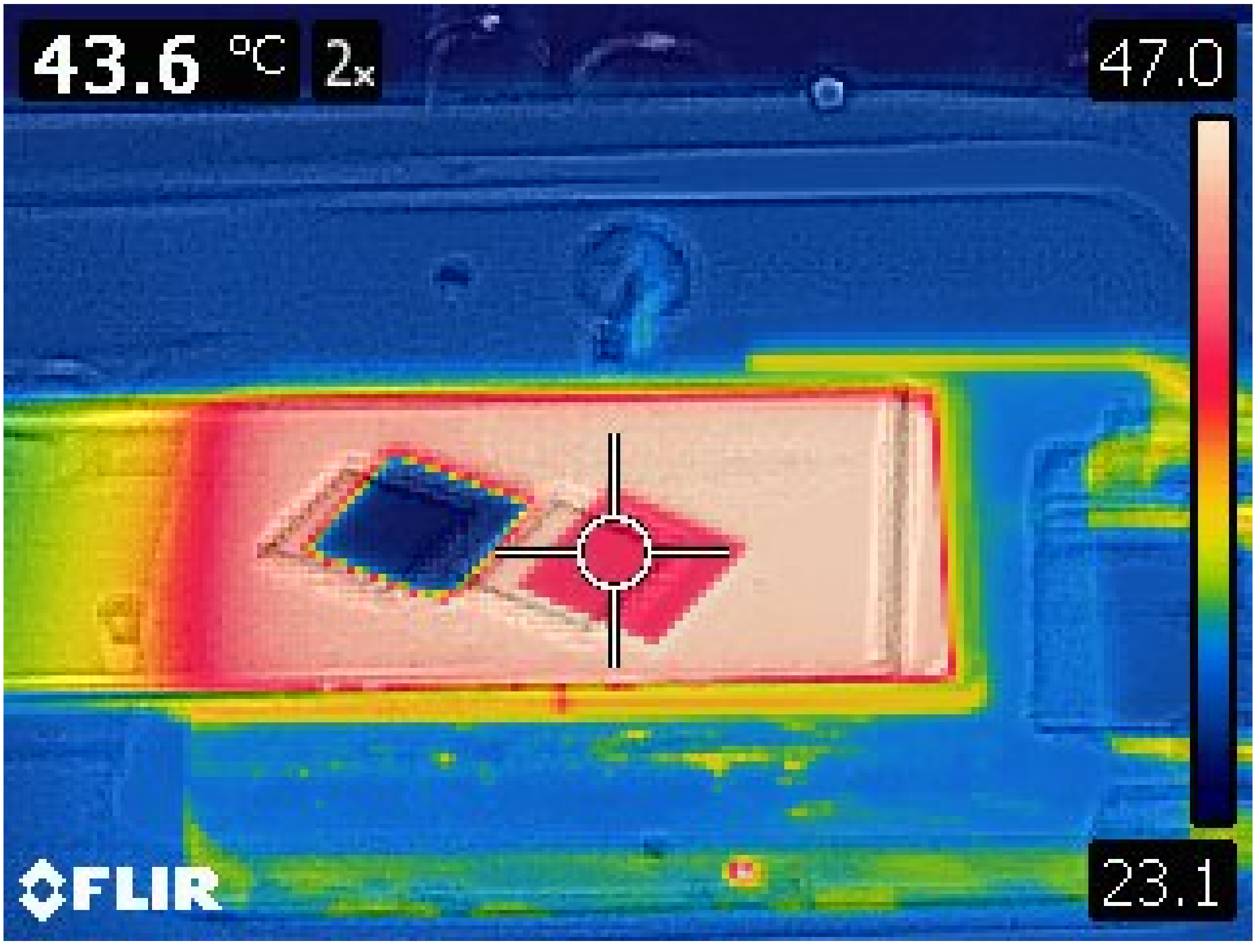

Figure S9: IR camera view at the sample stage heated to 50 °C. On the left is MIM sample (highly reflective, not possible to measure temperature), on the right - Si wafer piece unpolished side up (allowing to measure the temperature of approximately 44 °C).

4 Refractive index of PNIPAm hydrogel

The literature data[1] for the refractive index of PNIPAm hydrogel was confirmed with spectroscopic ellipsometry (with Woollam VASE ellipsometer). The real part of the refractive index of used PNIPAm-BP (48:1) hydrogel averaged across 4 samples with thicknesses of 84-99 nm is shown in **Figure S10**. In the modeling, we used the constant value of 1.503 for the refractive index, which corresponds to the experimental value at 650 nm wavelength. There was no noticeable difference between the spectra modeled with this constant value and with full dataset from the ellipsometry measurement. The imaginary part was measured as 0-0.02 across the studied region and was disregarded in our modeling.

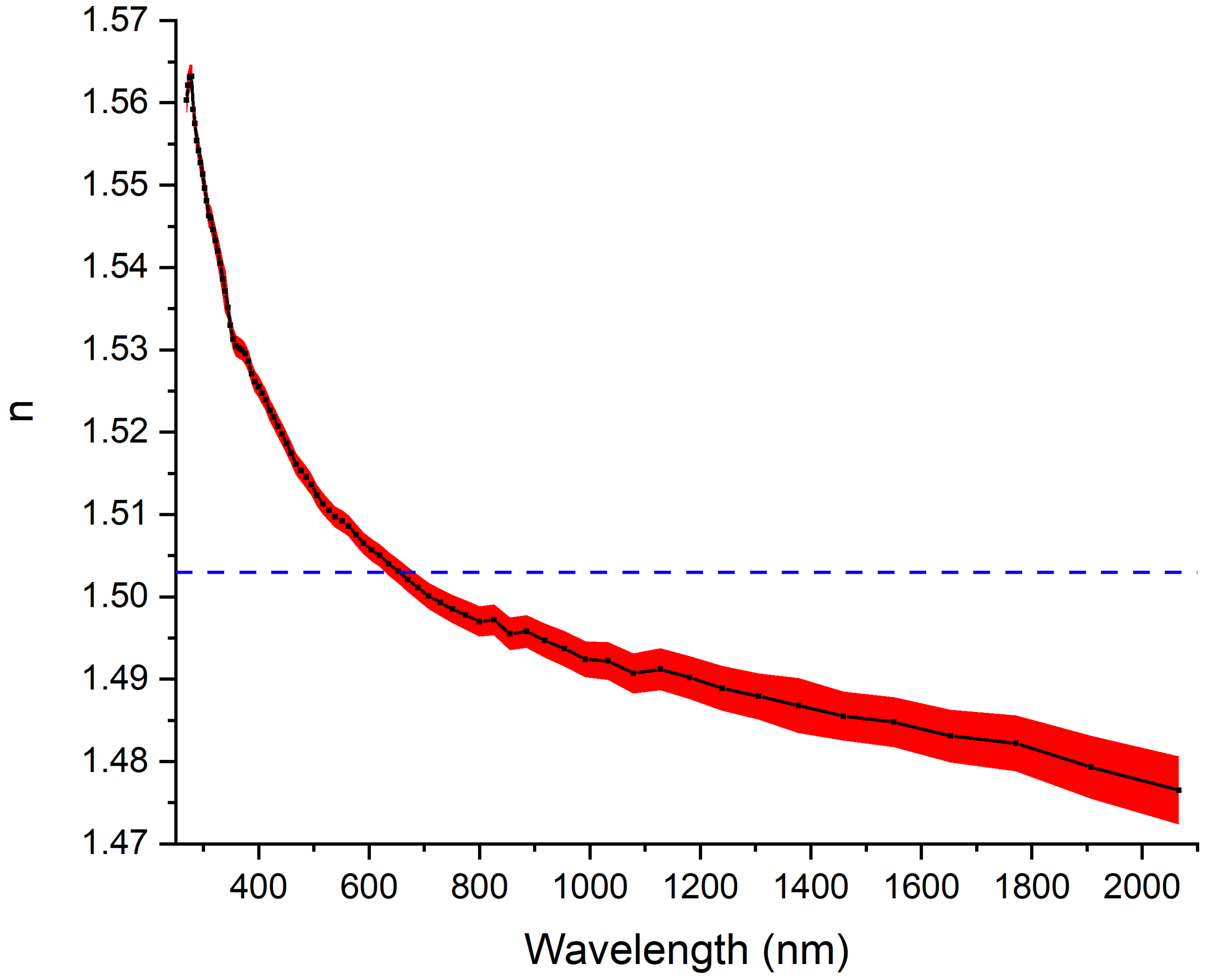

Figure S10: Real part of the refractive index of PNIPAm-BP hydrogel at ambient conditions (black), red area denotes the 90 % uncertainty interval. Blue dashed line corresponds to the average value of 1.503 used in the modeling.

References

(1) Brasse, Y.; Müller, M. B.; Karg, M.; Kuttner, C.; König, T. A. F.; Fery, A. Magnetic and Electric Resonances in Particle-to-Film-Coupled Functional Nanostructures. *ACS Appl. Mater. Interfaces* **2018**, *10*, 3133–3141.
